# Blood Lead Levels and Serum Insulin-Like Growth Factor 1 Concentrations in Peripubertal Boys

**DOI:** 10.1289/ehp.1206105

**Published:** 2013-04-26

**Authors:** Abby F. Fleisch, Jane S. Burns, Paige L. Williams, Mary M. Lee, Oleg Sergeyev, Susan A. Korrick, Russ Hauser

**Affiliations:** 1 Department of Endocrinology, Children’s Hospital Boston, Boston, Massachusetts, USA; 2 Department of Environmental Health, and; 3 Department of Biostatistics, Harvard School of Public Health, Boston, Massachusetts, USA; 4 Department of Pediatrics, and; 5 Department of Cell Biology, Pediatric Endocrine Division, University of Massachusetts Medical School, Worcester, Massachusetts, USA; 6 Department of Physical Education and Health, Samara State Medical University, Samara, Russian Federation; 7 Chapaevsk Medical Association, Chapaevsk, Russian Federation; 8 Channing Division of Network Medicine, Department of Medicine, Brigham and Women’s Hospital and Harvard Medical School, Boston, Massachusetts, USA

**Keywords:** cohort studies, growth, insulin-like growth factor 1, lead, puberty

## Abstract

Background: Childhood lead exposure has been associated with growth delay. However, the association between blood lead levels (BLLs) and insulin-like growth factor 1 (IGF-1) has not been characterized in a large cohort with low-level lead exposure.

Methods: We recruited 394 boys 8–9 years of age from an industrial Russian town in 2003–2005 and followed them annually thereafter. We used linear regression models to estimate the association of baseline BLLs with serum IGF-1 concentration at two follow-up visits (ages 10–11 and 12–13 years), adjusting for demographic and socioeconomic covariates.

Results: At study entry, median BLL was 3 μg/dL (range, < 0.5–31 μg/dL), most boys (86%) were prepubertal, and mean ± SD height and BMI *z*-scores were 0.14 ± 1.0 and –0.2 ± 1.3, respectively. After adjustment for covariates, the mean follow-up IGF-1 concentration was 29.2 ng/mL lower (95% CI: –43.8, –14.5) for boys with high versus low BLL (≥ 5 μg/dL or < 5 μg/dL); this difference persisted after further adjustment for pubertal status. The association of BLL with IGF-1 was stronger for mid-pubertal than prepubertal boys (*p* = 0.04). Relative to boys with BLLs < 2 μg/dL, adjusted mean IGF-1 concentrations decreased by 12.8 ng/mL (95% CI: –29.9, 4.4) for boys with BLLs of 3–4 μg/dL; 34.5 ng/mL (95% CI: –53.1, –16.0) for BLLs 5–9 μg/dL; and 60.4 ng/mL (95% CI: –90.9, –29.9) for BLLs ≥ 10 μg/dL.

Conclusions: In peripubertal boys with low-level lead exposure, higher BLLs were associated with lower serum IGF-1. Inhibition of the hypothalamic–pituitary–growth axis may be one possible pathway by which lead exposure leads to growth delay.

The Centers for Disease Control and Prevention (CDC) recently revised the reference value for childhood lead exposure downward from 10 to 5 μg/dL (Betts 2012). This has occurred in response to evidence showing consistent associations between blood lead levels (BLLs) < 10 μg/dL and neurocognitive (Bellinger et al. 1992; Lanphear et al. 2005), cardiovascular (Gump et al. 2005; Menke et al. 2006), and immunologic (Karmaus et al. 2005) outcomes, as well as pubertal (Kafourou et al. 1997; Naicker et al. 2010; Selevan et al. 2003; Shukla et al. 1991; Williams et al. 2010) and growth delay (Ballew et al. 1999; Little et al. 2009; Schwartz et al. 1986).

In cross-sectional studies conducted in prepubertal children, mean decreases of 1–1.5 cm in height have been estimated for each 10-μg/dL increase in BLL (Ballew et al. 1999; Little et al. 2009; Schwartz et al. 1986). BLL has also been negatively associated with height in two pubertal cohorts (Burns et al. 2012; Selevan et al. 2003). In our prospective cohort of Russian boys followed annually from 8 to 13 years of age, boys with BLL ≥ 5 μg/dL had significantly lower mean height *z*-score (–0.44) than boys with BLL < 5 μg/dL (Burns et al. 2012).

A mechanistic explanation for the association between lead exposure and growth delay in childhood has not been firmly established. Studies to this end have been conducted almost exclusively in animal models with high lead exposures, and results have suggested multiple possible mechanisms, perhaps operating simultaneously, including lead-induced reduction in food consumption (Hammond et al. 1989, 1990) and direct growth plate effects (Hass et al. 1967; Hicks et al. 1996). Several rodent studies have also implicated lead-mediated suppression of growth hormone (GH) release from the pituitary (Camoratto et al. 1993; Lau et al. 1991; Ronis et al. 1998a), and consistent with this finding, high lead exposure has been associated with decreased serum insulin-like growth factor 1 (IGF-1) concentration in rodents (Ronis et al. 1998a) and prepubertal children (Huseman et al. 1992).

The physiologic mechanisms that mediate health outcomes such as growth delay following low lead exposure may be different from those operating at higher exposures. Mechanistic differences in low- versus high-dose effects have been demonstrated for several environmental chemicals, including bisphenol A, atrazine, dioxin, and perchlorate (Vandenberg et al. 2012). Because exposures to lead and other heavy metals are increasingly regulated, a better understanding of the physiologic effects of low-level exposures is needed. With regard to growth delay, the relationship between low-level lead exposure and serum IGF-1 concentration has not been examined.

The present analysis was conducted to evaluate the association between childhood BLL and longitudinally measured serum IGF-1 concentrations and to assess whether there is a dose–response relationship between BLL and IGF-1 in a large cohort of peripubertal boys with low-level lead exposure.

## Methods

*Study population*. From 2003–2005, we recruited 8- to 9-year-old boys in Chapaevsk, Russia, to participate in the Russian Children’s Study, as previously described (Burns et al. 2012). Enrollment exclusion criteria included being institutionalized or having severe cerebral palsy. A total of 499 boys were thus identified, and they have been followed annually since recruitment. For the present analysis, 10 boys with severe chronic illnesses that could affect growth were excluded. Of the remaining 489, two pubertal boys were also excluded from the analysis because of implausibly low IGF-1 concentrations (< 50 ng/mL) despite normal height and body mass index (BMI) *z*-scores. Of the remaining 487 boys, 394 met inclusion criteria for this analysis, specifically, by availability of a baseline BLL (at 8–9 years) and follow-up serum IGF-1 concentration at both the 2-year (at 10–11 years) and the 4-year (at 12–13 years) follow-up visits. None of the participants had IGF-1 measured at baseline or BLL measured at follow-up. The Russian Children’s Study was approved by the human studies institutional review boards (IRBs) of the Chapaevsk Medical Association, Harvard School of Public Health, Brigham and Women’s Hospital, and University of Massachusetts Medical School. The parent or guardian of each Russian Children’s Study participant signed an informed consent form, and each boy signed an assent form. The present analysis was a secondary data analysis that was exempt from requirement for IRB review of already collected, deidentified data under federal and Children’s Hospital Boston policies.

*Study assessment protocol*. At study entry, boys underwent a physical examination and blood collection. We used a validated Russian Institute of Nutrition semiquantitative food-frequency questionnaire to estimate dietary intakes during the previous year (Martinchik et al. 1998; Rockett et al. 1997). Mothers or guardians also completed nurse-administered health and lifestyle questionnaires that included information on birth, family and child medical histories, occupational and residential history, and measures of socioeconomic status (SES) such as household income and parental education. We obtained birth weight and gestational age from medical records.

*Physical examination*. A single study nurse who did not have knowledge of the boys’ BLLs performed standardized anthropometric examinations, and a single investigator (O.S.) performed pubertal assessments at study entry and at annual follow-up visits. We measured height to the nearest 0.1 cm with a stadiometer. We measured weight to the nearest 100 g with a metric scale. We calculated age-adjusted percentiles for BMI (kilograms per meter squared) using the World Health Organization (WHO) standards (WHO 2011). For this analysis, pubertal status was based on testicular volume measured by Prader beads (orchidometer).

*Blood lead levels*. Venous blood samples (3.0 mL) were collected in trace metal-free Vacutainer tubes (Becton-Dickinson, Franklin Lakes, NJ, USA), after cleansing the venipuncture site with alcohol. Whole-blood samples were diluted with a matrix modifier solution and analyzed using Zeeman background corrected, flameless graphite furnace, atomic absorption spectrometry (ESA Laboratories, Chelmsford, MA, USA). BLLs below the limit of detection (1 μg/dL) were imputed as 0.5 μg/dL for 9 (2.2%) of 394 boys.

*Serum IGF-1 concentrations*. Serum IGF-1 concentrations were measured by a chemiluminescent immunometric assay using Siemens Immulite 2000 (Siemens AG, Munich, Germany). The assay is highly specific for IGF-1 with undetectable cross-reactivity with insulin, pro-insulin, luteinizing hormone (LH), thyroid-stimulating hormone, or insulin-like growth factor-2. The detection limit was 20 ng/mL; no IGF-1 values were below the limit of detection. The intra-assay coefficient of variation (CV) was < 3.9%, and the inter-assay CV was < 8.1% for the Immulite 2000 kit.

*Statistical analysis*. We used repeated measures analysis to estimate the association between BLL measured at 8–9 years of age and serum IGF-1 concentration at both the 2-year (at 10–11 years) and 4-year (at 12–13 years) follow-up visits. The distribution of BLLs was right-skewed with outliers. We considered several different ways of evaluating BLLs, including dichotomized as high (≥ 5 μg/dL) versus low (< 5 μg/dL) based on the new CDC threshold, as a continuous measure (log-transformed), and categorized as 0–2, 3–4, 5–9, or ≥ 10 μg/dL.

We fit linear regression models using a generalized estimating equation (GEE) approach to account for the repeated measures and slight skewness of IGF-1 concentrations. We first fit GEE linear regression models to evaluate unadjusted associations of high versus low BLL (≥ 5 or < 5 μg/dL) with serum IGF-1 concentrations. Next, we created a full multivariable model that included dichotomized BLL, birth weight (continuous), gestational age at birth (continuous), breastfeeding duration (< 12, 12–24, or > 24 weeks), maximum parental education (secondary education or less, junior college/technical training, or university graduate), monthly household income (< US$175, $175–250, or > $250), nutritional intake (total caloric intake and percent calories from protein, fat, and carbohydrate as continuous variables), baseline and follow-up age (continuous), and baseline and follow-up BMI [underweight (BMI < 10th percentile), overweight (BMI > 85th percentile), or normal weight]. We decided *a priori* not to consider height as a covariate due to its strong correlation with IGF-1 during puberty (Silbergeld et al. 1986). We then reduced this model by excluding covariates that did not predict the outcome with *p* ≤ 0.10, or did not change the estimated association between BLL and IGF-1 by > 10% when removed from the model, resulting in a final model that included baseline parental education, birth weight, nutritional intake, and baseline and follow-up age and BMI.

Because pubertal status is influenced by lead exposure (Selevan et al. 2003; Williams et al. 2010) and may be considered in the causal pathway between BLL and serum IGF-1 concentration, we fit the final reduced model both with and without adjustment for pubertal status [categorized based on testicular volume (TV) as prepubertal, ≤ 3 mL TV; early pubertal, > 3–6 mL TV; or mid-pubertal, > 6–15 mL TV]. No boys in this analysis had a TV > 15 mL. We also evaluated whether the association between BLL and IGF-1 concentration differed by pubertal stage by including an interaction term between pubertal status (prepubertal, early pubertal, or mid-pubertal) and BLL (≥ 5 or < 5 μg/dL).

Sensitivity analyses included the following modifications to the final reduced model: *a*) inclusion of maternal and paternal heights as potential confounding variables for the subset of boys who had these values available (*n* = 337), *b*) inclusion of 44 additional boys with only one follow-up IGF-1 concentration, *c*) BLL categorized as ≤ 2, 3–4, 5–9, or ≥ 10 μg/dL, and *d*) BLL modeled as a natural log-transformed continuous variable. We conducted all analyses using SAS version 9.3 (SAS Institute Inc., Cary, NC, USA), and we considered two-sided *p*-values ≤ 0.05 statistically significant.

## Results

Baseline growth and demographic characteristics of the 394 boys included are shown in Table 1. The 95 eligible boys excluded from the present analysis were not significantly different from those included with regard to the characteristics shown in Table 1, except that they tended to have a higher percent protein intake at baseline and were more likely to have parents that had junior college or technical training (data not shown).

**Table 1:** Baseline and follow-up characteristics of 394 boys from Chapaevsk, Russia, with baseline blood lead levels and two longitudinal measures of serum IGF-1.

Variable	8–9 years old (baseline)	10–11 years old	12–13 years old
Age (years) [median (range)]	8.1 (7.8–9.4)	10.1 (9.9–11.5)	12.1 (11.9–13.5)
BMI (WHO *z*-score)
Mean±SD	–0.2±1.3	–0.2±1.3	–0.2±1.4
≤10th percentile [*n* (%)]	67 (17)	83 (21)	75 (19)
>85th percentile [*n* (%)]	58 (15)	83 (21)	70 (18)
Height (WHO *z*-score) (mean±SD)	0.14±1.0	0.14±1.0	0.03±1.1
Testicular volume (mL) [*n* (%)]
≤3 (prepubertal)	336 (86)^*a*^	213 (54)	52 (13)^*a*^
>3–6	55 (14)	153 (39)	104 (27)
>6	0 (0)	28 (7)	235 (60)
IGF-1 (ng/mL) (mean±SD)		146.9±52.1	253.5±115.9
Birth weight (kg) (mean±SD)^*b*^	3.34±0.52
Gestational age (weeks) (mean±SD)^*a*^	39.01±1.74
Breastfeeding duration (weeks) [median (IQR)]^*c*^	13.0 (30.3)
Baseline nutritional intake (mean±SD)^*d*^
Total kcal/day	2,837±972
Percent fat	34.1±5.8
Percent protein	11.5±1.6
Percent carbohydrate	54.4±6.5
Monthly household income (US$) [*n* (%)]^*d*^
<175	136 (35)
175–250	107 (27)
>250	150 (38)
Maximal parental education [*n* (%)]^*a*^
Secondary education or less	25 (6)
Junior college/technical training	244 (62)
University graduate	122 (31)
Blood lead level (μg/dL)
Median (IQR)	3.0 (3.0)
<5 [*n* (%)]	285 (72)
≥5 [*n* (%)]	109 (28)
IQR, interquartile range. ^***a***^Five subjects missing. ^***b***^Two subjects missing. ^***c***^Five subjects missing. ^***d***^One subject missing.			

The median BLL at 8–9 years of age among boys included in the analysis was 3 μg/dL (25th, 75th percentiles: 2 μg/dL, 5 μg/dL; range, 0.5–31μg/dL). Most boys were prepubertal at baseline. The mean baseline height *z*-score was slightly above the WHO average, and the mean baseline BMI *z*-score was slightly below the WHO average (Table 1).

In unadjusted GEE models that accounted only for correlation among study visits, mean serum IGF-1 concentration during follow-up was 24.3 ng/mL lower (95% CI: –39.3, –9.3) among boys with BLL ≥ 5 μg/dL compared with those with < 5 μg/dL. A significantly lower mean IGF-1 concentration for boys with high versus low BLL was also estimated based on the full multivariable model (–28.0 ng/mL; 95% CI: –43.1, –12.9) and the final reduced model (–29.2 ng/mL; 95% CI: –43.8, –14.5) (Table 2). When pubertal status was added to the final reduced model, the association of high BLL with IGF-1 concentration was modestly attenuated (adjusted mean difference = –24.4 ng/mL; 95% CI: –37.7, –11.1).

**Table 2:** Repeated measures generalized estimating equation models predicting the mean levels of serum concentrations of IGF-1 (ng/mL) in relation to blood lead levels and relevant covariates.

Covariate	Full multivariable model(*n*=385 boys, 767 visits)		Final reduced model(*n*=389 boys, 775 visits)	
	Adjusted mean change (95% CI)	*p*-Value	Adjusted mean change(95% CI)	*p*-Value
Lead (μg/dL)
<5	Reference		Reference
≥5	–28.0 (–43.1, –12.9)	<0.001	–29.2 (–43.8, –14.5)	<0.001
Age (years)	51.9 (47.2, 56.6)	<0.001	52.1 (42.4, 56.8)	<0.001
Birth weight (kg)	–17.4 (–33.2, –1.5)	0.03	–17.5 (–31.5, –3.5)	0.01
BMI *z*-score (percentile)
≤10	–58.6 (–73.9, –43.3)	<0.001	–61.3 (–76.7, –45.8)	<0.001
>10–85	Reference		Reference
>85	13.8 (–4.4, 31.9)	0.14	12.9 (–5.2, 30.9)	0.16
Nutritional intake
Total calories^*a*^	–2.6 (–10.7, 5.6)	0.54	–2.6 (–10.6, 5.5)	0.54
Fat (percent)	1.4 (0.2, 2.6)	0.02	1.3 (0.1–2.5)	0.03
Protein (percent)	3.0 (–1.6, 7.6)	0.21	3.1 (–1.4, 7.6)	0.18
Parental education
Secondary education or less	–22.5 (–45.8, 0.8)	0.06	–24.9 (–47.7, –2.0)	0.03
Junior college/technical training	–3.4 (–19.3, 12.5)	0.69	–2.7 (–18.4, 13.0)	0.74
University graduate	Reference		Reference
Monthly household income (US$)
<175	–3.3 (–20.3, 13.8)	0.71
175–250	–6.4 (–23.7, 10.9)	0.47
>250	Reference
Gestational age (weeks)	0.4 (–4.2, 5.1)	0.85
Breastfeeding (weeks)
<12	Reference
12–24	0.7 (–18.2, 19.7)	0.94
>24	1.1 (–14.1, 16.4)	0.88
^***a***^Per 1,000 calories.				

**Figure 1:**
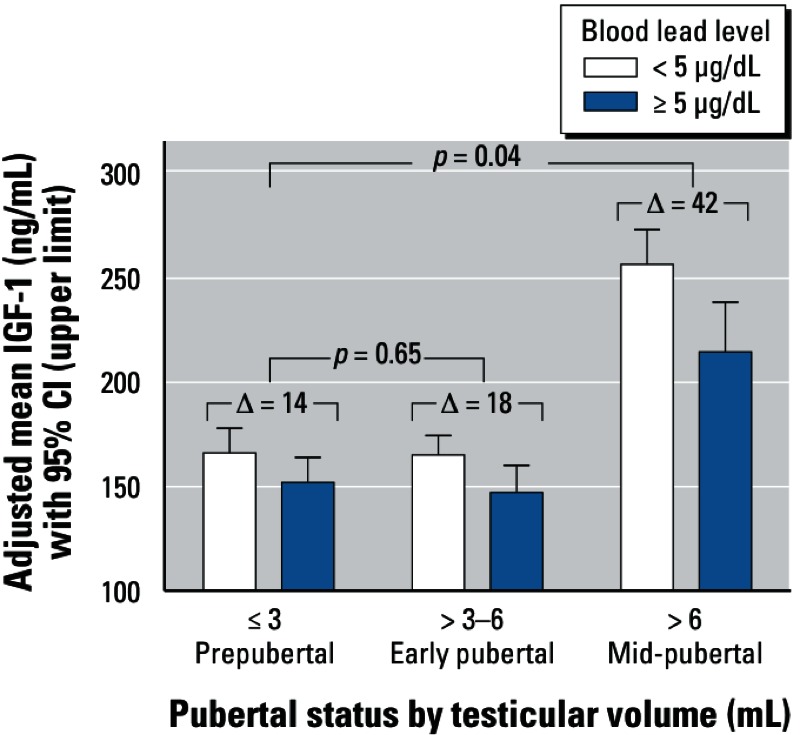
Adjusted mean IGF-1 concentrations for high versus low BLL by pubertal status. The adjusted mean IGF-1 difference for high versus low BLL was 14 ng/mL in prepubertal boys, 18 ng/mL in early-pubertal boys, and 42 ng/mL in mid-pubertal boys. Compared with prepubertal boys, the lead-associated IGF-1 difference was larger in mid-pubertal boys (*p* = 0.04) and larger, but not significantly larger, in early-pubertal boys (*p* = 0.65).

The association of BLL with IGF-1 concentration differed according to pubertal status. In particular, the reduction in adjusted mean IGF-1 concentrations between high versus low BLL groups was greater among boys in mid-puberty than for prepubertal boys (–41.9 ng/mL; 95% CI: –15.1, –68.7 vs. –14.1 ng/mL; 95% CI: –0.9, –27.2; interaction *p*-value = 0.04). The reduction in adjusted mean IGF-1 concentrations between high versus low BLL groups was slightly larger for boys in early puberty (–18.0 ng/mL; 95% CI: –3.5, –32.5) than for prepubertal boys (interaction *p*-value = 0.64) (Figure 1). Adjusted mean percent decreases in IGF-1 concentrations between high versus low BLL groups were 9.3%, 12.2%, and 19.5% for prepubertal boys, boys in early puberty, and boys in mid-puberty, respectively.

In sensitivity analyses, further adjustment for parental heights among the subset of boys (*n* = 337) with these measures available had no appreciable impact on the estimated difference in IGF-1 concentrations for boys with high versus low BLL (adjusted mean difference = –31.6 ng/mL; 95% CI: –48.2, –15.0). The estimated difference in IGF-1 was also similar based on a model that included follow-up IGF-1 concentrations for 438 boys with at least one IGF-1 measurement (adjusted mean difference = –28.8 ng/mL; 95% CI: –42.5, –15.1).

**Figure 2:**
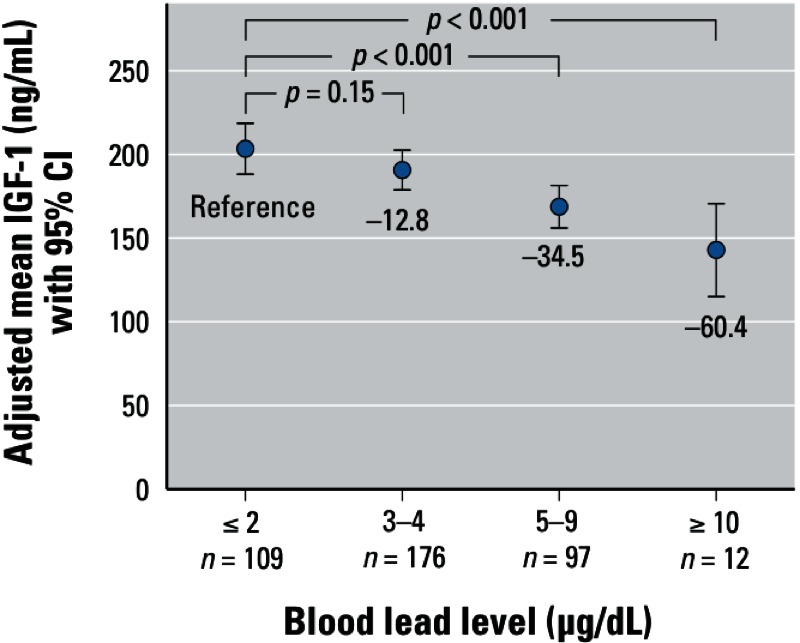
Adjusted mean IGF-1 concentrations by BLL category. Compared with BLL ≤ 2 μg/dL, the higher BLL levels of 5–9 μg/dL and ≥ 10 μg/dL were associated with significantly lower mean IGF-1 concentrations (*p* < 0.001 for both comparisons).

When BLL was divided into finer categories, adjusted mean IGF-1 concentrations decreased monotonically relative to the reference group: –12.8 ng/mL (95% CI: –29.9, 4.4; *n* = 176) for BLL = 3–4 μg/dL; –34.5 ng/mL (95% CI: –53.1, –16.0; *n* = 97) for BLL 5–9 μg/dL; and –60.4 ng/mL (95% CI: –90.9, –29.9; *n* = 12) for BLL ≥ 10 μg/dL, compared with BLL ≤ 2 μg/dL (*n* = 109) (Figure 2). Finally, each unit increase in natural log-transformed BLL was associated with a 22.2-ng/mL decrease in mean serum IGF-1 (95% CI: –33.9, –10.6).

## Discussion

We observed a negative association between BLLs measured at 8–9 years of age and serum IGF-1 concentrations at 10–11 and 12–13 years of age that was stronger among boys in mid-puberty than in prepubertal boys. This finding suggests one possible explanation for our previous finding of lower mean height *z*-scores for boys in the same cohort with BLL ≥ 5 μg/dL compared with BLL < 5 μg/dL (Burns et al. 2012). Results from the present analysis suggest a montonic dose–response relationship between BLL and serum IGF-1 that adds further support to existing evidence of physiological effects of BLLs < 10 μg/dL (Ballew et al. 1999; Bellinger et al. 1992; Gump et al. 2005; Kafourou et al. 1997; Karmaus et al. 2005; Lanphear et al. 2005; Little et al. 2009; Menke et al. 2006; Naicker et al. 2010; Selevan et al. 2003; Shukla et al. 1991; Williams et al. 2010).

A negative association between BLL and serum IGF-1 concentrations is consistent with lead-induced inhibition of the hypothalamic–pituitary–growth axis. An *in vitro* study in rat pituitary demonstrated that lead blocked the binding of growth hormone releasing hormone to its receptor, suggesting axis inhibition at the level of the pituitary (Lau et al. 1991). Lead could also affect the growth axis at the level of the pituitary through interference with calcium-dependent GH release. In *in vitro* studies of bovine and rat pituitary, other divalent cations such as zinc, nickel, cadmium, and magnesium blocked calcium-dependent GH release (Carlson 1984; Lorenson et al. 1983), although additional studies are needed to determine whether this effect persists in *in vivo* and whether lead elicits similar actions. Consistent with these potential pituitary-mediated effects, a study of rodent pups (mean BLL, 18 µg/dL) demonstrated suppressed growth hormone releasing hormone-stimulated GH release (Camoratto et al. 1993), and in a separate rodent model, high lead exposure (up to mean BLL of 263 µg/dL) resulted in lower serum IGF-1 concentrations (Ronis et al. 1998a). Highly lead-exposed prepubertal children (*n* = 6) had decreased mean 24-hr serum GH concentrations and lower serum IGF-1 concentrations before chelation (BLLs > 40 µg/dL) compared with after chelation (BLLs ≤ 30 µg/dL) (Huseman et al. 1992). In contrast to these studies, the present analysis explored BLLs within currently acceptable ranges and still found a negative association between BLL and IGF-1.

Lead may also inhibit the reproductive axis at the level of the pituitary. Six men with occupational lead exposure (mean BLL, 38.7 µg/dL) had blunted LH response to gonadotropin-releasing hormone (GnRH) compared with nine men without occupational exposure (mean BLL, 16 µg/dL) (Braunstein et al. 1978). In a study of 77 lead smelter workers (mean BLL, 33 µg/dL) and 26 nonworkers (mean BLL, 4.1 µg/dL), a subset analysis demonstrated lower GnRH-stimulated follicle-stimulating hormone in 9 workers compared to 11 nonworkers (Erfurth et al. 2001). Also, lead exposure in rodents resulted in decreased serum LH concentration (Ronis et al. 1998b) and increased pituitary LH stores (Klein et al. 1994; Sokol et al. 1998), further suggesting possible pituitary hyporesponsiveness to GnRH. Consistent with these studies of gonadotropin inhibition, lead exposure has been associated with later onset of puberty in our cohort (Williams et al. 2010) and in other adolescent cohorts (Naicker et al. 2010; Selevan et al. 2003).

A lead-induced decrement in IGF-1 may contribute to gonadotropin inhibition and pubertal delay. Specifically, IGF-1 has been shown to activate GnRH *in vitro* (Zhen et al. 1997) and in rodent models (Hiney et al. 1991, 1996), and puberty is delayed in GnRH-specific IGF-1 receptor knock-out mice (Divall et al. 2010). Furthermore, IGF-1 administration to lead-exposed mice with delayed puberty restored pubertal timing (Pine et al. 2006), providing additional evidence for a potential mediating role of IGF-1 in the association between lead exposure and delayed puberty.

In addition to inhibition of GH and gonadotropin release, in animal studies, high lead exposure has been associated with other processes that could lead to growth delay such as decreased food consumption (Hammond et al. 1989, 1990) and reduced formation of new bone (Hass et al. 1967; Hicks et al. 1996). Future studies should explore whether these effects can be observed in humans with low-level lead exposures.

As far as we are aware, our study is the first to identify puberty as a particularly vulnerable period in which to assess lead’s effect on IGF-1. Both the absolute and percent decrease in IGF-1 in association with lead exposure was larger in mid-pubertal boys than in prepubertal or early-pubertal boys. Thus, in our cohort, puberty seemed to be a key time period in which to detect an effect of lead, and this may be generalizable to other environmental epidemiologic studies examining outcomes of growth and associated hormones.

The present study is limited by availability of the BLL measurement at only one time point, leading to an inability to explore other vulnerable windows of exposure, such as exposures during infancy that may have a stronger association with childhood height (Afeiche et al. 2012). Also, none of the participants had a baseline IGF-1 measurement. However, we believe that a prospective evaluation of BLL on subsequent IGF-1 values made for a stronger study design.

Future studies of lead and growth would benefit from measurement of serum insulin-like growth factor-binding protein 3, a less nutritionally dependent measure of GH activity. Inclusion of girls in future studies will also be important, because rodent models suggest that lead’s effect on pubertal growth may be more pronounced in males than in females (Ronis et al. 1998a). Furthermore, the net effect of lead on growth in humans cannot be completely understood without information on the association between childhood lead exposures and adult height, so continued longitudinal follow-up through adulthood is warranted for this and other cohorts.

## Conclusion

In the present study we found a negative monotonic dose–response association between blood lead levels in boys at 8–9 years of age and their serum IGF-1 concentrations at 10–11 and 12–13 years of age. With increasing attention to environmental exposures and potential health risks, it is essential to better understand effects of low-level lead exposure on key developmental processes such as growth and reproductive development.
